# Association between Temporomandibular Joint Disorder and Parkinson’s Disease

**DOI:** 10.3390/brainsci11060747

**Published:** 2021-06-04

**Authors:** Hyo-Geun Choi, Joo-Heung Yoon, Tae-Hwan Chung, Chanyang Min, Dae-Myoung Yoo, Jee-Hye Wee, Suk-Yun Kang, Yeso Choi, Seok-Jin Hong, Soo-Hwan Byun

**Affiliations:** 1 Hallym Data Science Laboratory, College of Medicine, Hallym University, Anyang 14068, Korea; pupen@naver.com (H.-G.C.); joicemin@naver.com (C.M.); ydm1285@naver.com (D.-M.Y.); weejh07@hanmail.net (J.-H.W.); 2 Department of Otorhinolaryngology–Head and Neck Surgery, College of Medicine, Hallym University, Anyang 14068, Korea; 3 Research Center of Clinical Dentistry, Clinical Dentistry Graduate School, Hallym University, Chuncheon 24252, Korea; enthsj@hanmail.net; 4 Department of Medicine, University of Pittsburgh, Pittsburgh, PA 15260, USA; yoonjh@upmc.edu; 5 Department of Physical Medicine and Rehabilitation, Johns Hopkins University, Baltimore, MD 21218, USA; tchung7@jhmi.edu; 6 Department of Neurology, College of Medicine, Hallym University, Dongtan 18450, Korea; sukyunkang@hallym.or.kr; 7 Department of Otorhinolaryngology–Head and Neck Surgery, College of Medicine, Hallym University, Dongtan 18450, Korea; 201080@hallym.or.kr; 8 Department of Oral and Maxillofacial Surgery, Dentistry, College of Medicine, Hallym University, Anyang 14068, Korea

**Keywords:** temporomandibular joint disorder, Parkinson’s disease, jaw movement

## Abstract

This study performed two different analyses using a large set of population data from the Korean National Health Insurance Service Health Screening Cohort to evaluate the interactional association between temporomandibular disorder (TMD) and Parkinson’s disease (PD). Two nested case–control population-based studies were conducted on 514,866 participants. In Study I, 4455 participants with TMD were matched with 17,820 control participants, with a ratio of 1:4. In Study II, 6076 participants with PD were matched with 24,304 control participants, with a ratio of 1:4. Obesity, smoking, alcohol consumption, systolic, diastolic blood pressure, fasting blood glucose level, and total cholesterol were adjusted. The adjusted odds ratio (OR) for TMD was 1.43 (95% confidence interval (CI) = 1.02–2.00) in PD patients compared to non-PD patients in Study I (*p* < 0.001). The adjusted OR for PD was 1.56 (95% CI = 1.13–2.15) in TMD patients compared to non-TMD patients in Study II (*p* = 0.007). This study demonstrated that patients with TMD have a significantly higher risk of developing PD and, conversely, those with PD have a significantly higher risk of developing TMD.

## 1. Introduction

The American Academy of Orofacial Pain defines temporomandibular disorder (TMD) as a clinical condition involving the temporomandibular joint (TMJ), masticatory muscles, and related anatomic structures [1]. Limitations of masticatory function, local pain, noises, and deviations of the mandible are typical symptoms and signs of TMD [2]. TMD is common among adolescents and adults. Approximately 5–70% of the general population presumably has TMD [3,4]. More specifically, studies have reported that the prevalence rate of TMD is 2–5% in adolescents and 25–52% in adults, with the female population having a higher prevalence than that of the male population [5,6,7]. TMD occurs in up to 14% of females and 10% of males [8]. TMD is associated with various diseases, including migraine, rheumatoid arthritis, osteomyelitis, and psoriatic arthritis [9,10,11,12]. Byun et al. demonstrated that TMD patients have a higher risk of migraines. These results suggest that dentists can reduce the risk of migraines in TMD patients through proper management [12]. Another study reported that those with rheumatic arthritis have a higher risk of TMD and should be monitored for early symptoms of TMD to prevent the risk of aggravation [11].

Some studies suggested that the research diagnostic criteria of TMD are divided into three physical diagnostic categories. First, masticatory muscle pain is considered myofascial TMD with or without limited jaw opening. Second, TMJ disc displacement is defined as reducing or non-reducing with or without limited jaw opening. Third, other joint conditions consist of arthralgia, arthritis, and arthrosis [9,13,14,15,16]. Many patients are diagnosed to have TMD with limited mouth opening or pain around the TMJ and masticatory muscle area [17].

TMD pain could originate from either the TMJ or the myofascia. In TMJ, excessive loading through local hypoxia, increased inflammatory mediators, and stimulation of nociceptors can cause pain, as well as degenerative changes. Increased nociceptive input from a painful TMJ would lead to prolonged neuron sensitization in the central nervous system, which in turn could lower TMJ pain thresholds and tolerance [18]. The pathophysiology of myofascial TMD is much less elucidated. There is no definite evidence that the masticatory muscle tone is activated to a level that would induce painful muscle spasm. Muscle pain could decrease jaw mobility and thus may result in a protective response. Parafunctional activities such as clenching could cause consistent strain injuries to the muscle. This phenomenon may induce pain in the masticatory muscles by the induction of localized tissue ischemia or release of algogenic substances such as serotonin or glutamate to sensitize and activate muscle nociceptors [18]

Parkinson’s disease (PD) is the second most common neurodegenerative disorder, affecting 7 million people worldwide [19]. PD was initially considered as commonly manifesting tremor (shaking), bradykinesia (slowness), and rigidity (stiffness). Much published evidence suggests that PD is a very complex, multisystem disorder of motor and non-motor syndrome [20]. Various studies have reported associations between PD and other diseases [21,22,23,24,25]. Chen et al. demonstrated the potential association of intestinal inflammation with PD pathogenesis [22]. Increased intestinal permeability caused by gut inflammation induces the leakage of flora and its metabolites into the body [22].

The pathophysiology of PD is also poorly understood, but is considered to be influenced by both genetic and environmental factors [26]. Thus, PD is not simply a destructive disorder initiated by the degeneration of dopaminergic neurons in the substantia nigra, resulting in dysfunctioning motor symptoms [21], which is commonly based on signs and symptoms, requiring examinations such as neuroimaging to rule out other diseases [26].

A previous study reported that PD patients showed a higher risk of bruxism due to the progression of motor symptoms, and patients with bruxism are more likely to be affected by TMD [27]. Another study suggested that muscle rigidity in PD could be associated with higher tone and elevated muscle tone, possibly including the masticatory muscle [24]. Elevated sleep muscle tone has been detected in the masticatory muscles of TMD patients. In addition, awake tooth-to-tooth contact might also cause a mild increase in masticatory muscle tone activity in TMD patients.

A few studies have shown a significant relationship between TMD and PD in terms of physical inability, including its negative impact on the physical activities of daily life [23,28]. However, Silva et al. [28] used a cross-sectional design with a small sample size. Chen et al. [23] only estimated the risk of TMD in PD patients and thereby did not evaluate the risk of PD in TMD patients. Moreover, these studies focused only on the prevalence of symptoms and signs [23,28].

This study aimed to explore the association between TMD and PD in a large population cohort by conducting two nested case–control studies. In this study, two different evaluations were performed using data from the Korean National Health Insurance Service Health Screening Cohort.

In the first evaluation (Study I), it was hypothesized that TMD patients would have a significantly higher risk of PD than the matched non-TMD patients. In the second evaluation (Study II), it was hypothesized that PD patients would have a significantly higher risk of TMD than the matched non-PD patients.

## 2. Methods

### 2.1. Study Population

This study obtained approval from the ethics committee of Hallym University (2019-10-023). Informed consent was examined by the Institutional Review Board. All analyses were in accordance with the guidelines and regulations of the ethics committee of Hallym University. A more specific explanation of the Korean National Health Insurance Service Health Screening Cohort data can be found in previous studies [29,30].

### 2.2. Definition of Temporomandibular Joint Disorder

TMD has been defined as having a diagnosis of ICD-10 code K07.6 (temporomandibular joint disorders). To enhance the accuracy of the diagnosis, this study included participants who were treated twice or more for TMD.

### 2.3. Definition of Parkinson’s Disease

PD has been defined as having a diagnosis of ICD-10 code G20 (Parkinson’s disease). To enhance the accuracy of the diagnosis, this study included participants who were treated twice or more for PD.

### 2.4. Participant Selection

#### 2.4.1. Study I

**Figure 1:**
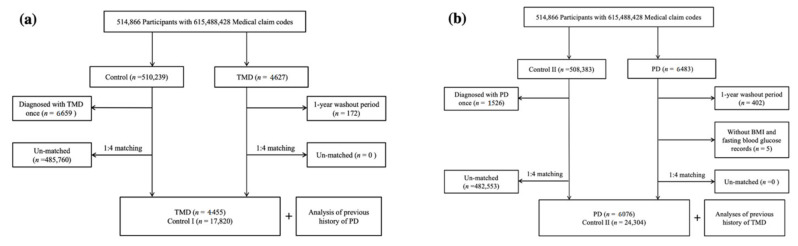
A schematic illustration of the participant selection process: (**a**) TMD in PD; (**b**) PD in TMD. TMD, temporomandibular joint disorder; PD, Parkinson’s disease.

Study I selected participants with TMD from 514,866 participants with 615,488,428 medical claim codes from 2002 to 2015 (*n* = 4627). The control group consisted of participants who were not identified to have TMD from 2002 to 2015 (*n* = 510,239). Participants with TMD who had a 1-year washout period were excluded from the study (*n* = 172). In the control group, participants were excluded if they had been diagnosed with TMD (*n* = 6659). Participants with TMD were matched with control participants adjusted for age, sex, income, and region of residence, with a ratio of 1:4. The control participants were selected randomly in order to reduce selection bias. The index date of each participant with TMD was decided as the time of treatment for TMD. The index date of the control participants was decided as the index date of their matched participant with TMD. Therefore, each matched participant with TMD had the same index date as the control participants. During the matching process, 485,760 control participants were excluded. Finally, 4455 participants with TMD were matched with 17,820 control I participants, with a ratio of 1:4 (Figure 1a). If a participant developed PD after the index date, then he or she was not considered to be a participant with PD in this study.

#### 2.4.2. Study II

Study II selected 6482 participants with PD from 514,866 participants with 615,488,428 medical claim codes from 2002 to 2015. The control group (*n* = 508,383) consisted of participants who were not diagnosed with PD from 2002 to 2015. Participants with PD who had a 1-year washout period were excluded from the study (*n* = 402). Among the participants with PD, those lacking body mass index (BMI) and fasting blood glucose data were excluded (*n* = 5). In the control group, participants were excluded if they had been diagnosed with PD once (*n* = 1526). Participants with PD were matched with control participants adjusted for age, sex, income, and region of residence, with a ratio of 1:4. The control participants were selected randomly in order to reduce selection bias. The index date of each participant with PD was decided as the time of treatment for PD. The index date of control participants was decided as the index date of their matched participants with PD. Therefore, each matched participant with PD had the same index date as the control participants. During the matching process, 482,553 control participants were excluded. Finally, 6076 participants with PD were matched with 24,304 control II participants, with a ratio of 1:4 (Figure 1b). If a participant developed TMD after the index date, then he or she was not considered to be a participant with TMD in this study.

### 2.5. Covariates

The age groups were categorized into 5-year intervals from age 40 to 85, with one other group for those aged >85 years (10 age groups in total). Income groups were divided into five classes (class 1—lowest income to class 5—highest income). The region of residence was divided into rural and urban areas, in accordance with our previous study [31].

Tobacco smoking was decided based on the participant’s current smoking status (non-smoker, current smoker, and past smoker). Alcohol consumption was categorized on the basis of the frequency of alcohol consumption (once a week or less and twice a week or more). Obesity was measured using the BMI (kg/m^2^). The BMI was categorized as underweight (<18.5), normal (≥18.5 to <23), overweight (≥23 to <25), obese grade I (≥25 to <30), and obese grade II (≥30) based on the Asia Pacific criteria following the Western Pacific Regional Office 200 [32,33]. Systolic blood pressure, diastolic blood pressure, fasting blood glucose, and total cholesterol were measured.

The Charlson comorbidity index (CCI) has been applied widely for the measurement of disease burden using 17 comorbidities. CCI was measured as the continuous variable (0—no comorbidities to 29—multiple comorbidities) [34].

### 2.6. Statistical Analyses

The general characteristics between the TMD and control I groups in Study I and PD and control II groups in Study II were compared using the chi-square test.

To analyze the odds ratios (ORs) with 95% confidence intervals (CIs), a conditional logistic regression model was used for TMD in PD and control I groups in Study I and for PD in TMD and control II groups in Study II. In these analyses, the crude model and the model adjusted for smoking, obesity, alcohol consumption, fasting blood glucose, systolic blood pressure, diastolic blood pressure, total cholesterol, and CCI scores were devised. The analysis was stratified for matching variables such as age, sex, income, and region of residence.

For the subgroup analyses in Study I and Study II, we divided the participants by age and sex (<60 years old and ≥60 years old, male and female; <70 years old and ≥70 years old, male and female), respectively, and analyzed the crude and the adjusted models.

In the present study, additional subgroup analyses were performed using the unconditional logistic regression model. We stratified the participants by obesity (BMI < 23 and BMI ≥ 23), smoking status (non-smoker and smoker), alcohol consumption (once a week or less and twice a week or more), blood pressure (normal and hypertension), fasting blood glucose (<100 mg/dL and ≥100 mg/dL), and total cholesterol (<200 mg/dL and ≥200 mg/dL), respectively. In these analyses, the crude model and the model adjusted for obesity, smoking, alcohol consumption, systolic blood pressure, diastolic blood pressure, fasting blood glucose, total cholesterol, and CCI scores were devised.

Two-tailed analyses were performed, and significance was defined as *p*-values < 0.05. The SAS version 9.4 software (SAS Institute Inc., Cary, NC, USA) was used for statistical analyses.

## 3. Results

The rates of age, sex, income, and region of residence were exactly the same between TMD and control I and PD and control II due to the matching (Table 1). The other general characteristics were different between them.

The adjusted OR for TMD was 1.43 (95% CI = 1.02–2.00) in PD compared to non-PD in Study I (*p* < 0.001, Table 2).

**Figure 2:**
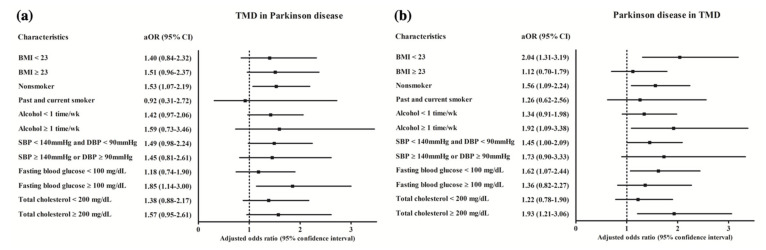
Subgroup analyses of (**a**) TMD in PD and (**b**) PD in TMD. TMD, temporomandibular joint disorder; PD, Parkinson’s disease.

In subgroup analyses according to age and sex, the results did not reach the statistical significance. In other subgroup analyses according to income and region, the results reached statistical significance only in high income group (adjusted OR = 1.80, 95% CI = 1.08–3.00, *p* = 0.025). This was consistent in subgroup of non-smoker (adjusted OR = 1.53, 95% CI = 1.07–2.19, *p* = 0.019) and fasting blood glucose ≥100 mg/dL (adjusted OR = 1.85, 95% CI = 1.14–3.00, *p* = 0.013) (Table S1 and Figure 2a).

The adjusted OR for PD was 1.56 (95% CI = 1.13–2.15) in TMD compared to non-TMD in Study II (*p* = 0.007, Table 3). In subgroup analyses, only ≥70 years old women reached statistical significance (adjusted OR = 1.68, 95% CI = 1.04–2.70, *p* = 0.033). In other subgroup analyses according to income and region, the adjusted OR was 1.66 (95% CI = 1.01–2.73, *p* = 0.046) in high income, and 1.65 (95% CI = 1.12–2.43, *p* = 0.011) in rural living. This was consistent in subgroup of BMI < 23 (adjusted OR = 2.04, 95% CI = 1.31–3.19, *p* = 0.002), non-smoker (adjusted OR = 1.56, 95% CI = 1.09–2.24, *p* = 0.015), alcohol consumption ≥ once a week (adjusted OR = 1.92, 95% CI = 1.09–3.38, *p* = 0.024), without hypertension (adjusted OR = 1.45, 95% CI = 1.00–2.09, *p* = 0.048), fasting blood glucose < 100 mg/dL (adjusted OR = 1.62, 95% CI = 1.07–2.44, *p* = 0.022), and total cholesterol ≥ 200 mg/dL (adjusted OR = 1.93, 95% CI = 1.21–3.06, *p* = 0.006) (Table S2 and Figure 2b).

## 4. Discussion

The present study analyzed two different study designs using the Korean National Health Insurance Service Health Screening Cohort data. This study investigated both the risk of PD in TMD patients and the risk of TMD in PD patients. Thus, both of these nested case–control studies are different from previous studies [23,28]. This is the novelty of the present study.

Study I demonstrated that TMD patients had a significantly higher risk of PD than the matched non-TMD patients (Table 2). Merel et al. reported that patients with PD reported significantly more bruxism when asleep as well as awake than those in control groups. In addition, in the same study, patients with PD had more TMD symptoms and reported a significantly higher mean pain intensity in the orofacial region than those in the non-PD population [35].

Patients with PD commonly experience stiffness and tremors, symptoms which could induce bruxism or clenching [35]. Laine et al. reported that bruxism is associated with abnormal jaw tremor and that its assessment may aid in the detection of bruxism [36]. Bruxism is a common behavior related to unconscious jaw movement, which can lead to pain, headache, severe dental damage, and TMD [37]. This condition may be induced by a malfunction in the recognition of afferent feedback from the teeth and jaw [38]. Patients with bruxism may not be able to estimate the appropriate bite force for chewing or biting, which indicates a sensorimotor deficit with uncontrollable jaw force [39]. In addition, the tremor or rigidity characteristic of PD could be associated with bruxism, which could aggravate TMD problems [40]. Verhoeff et al. reported that bruxism is a common condition in PD patients, but it is not associated with the dopaminergic medication dose [40].

Another study revealed that elevated muscle tone is associated with PD [24]. Compared with the group without PD, that with the disease showed an increase in electromyographic fatigue, with significant differences for the right masseter and right temporal muscles. The results suggest a link between PD and functional alterations of the masticatory system. Tan et al. reported that severe tonic spasms of the masseter muscles activate jaw closure and lead to tooth fracture in patients with PD [41]. Rhythmic and repeated masticatory muscle activity was identified during non-rapid eye movement sleep in PD patients with rapid eye movement sleep behavior disorder [42].

Study II showed that PD patients had a significantly higher risk of TMD than the matched non-PD patients (Table 3). In other words, many participants with PD had TMD prior to the diagnosis of PD. In particular, the diagnosis of TMD prior to the diagnosis of PD was more common in PD patients than in participants without PD. Recently, another study reported that PD patients had an increased risk of TMD compared to the matched controls, with the difference being significant for 2 years after the diagnosis of PD in Taiwan’s population [23].

Jaw or chin tremors may occur in neurodegenerative disease, which could be an early sign of PD [43,44]. Rossi et al. reported that facial tremor (jaw or chin) in patients, with or without associated parkinsonism lasting less than 1 year at the time of the acute levodopa challenge, showed 92% sensitivity and 93% specificity in the prediction of a final PD diagnosis [44]. Udagedara et al. explained that the presence of acute-onset jaw tremor and its dramatic response to levodopa in the presence of unilateral infarction could be associated with vascular PD [45]. As mentioned previously, jaw tremor could be associated with bruxism and TMD [36]. Jaw tremor, as an initial manifestation of PD, could induce bruxism and aggravate TMD prior to the diagnosis of PD. This mechanism could possibly explain the result of Study II. Decreased jaw mobility or jaw tremor at the initial stage of PD worsens the masticatory and maxillofacial functions [25]. Dentists should be cautious of the possibility of jaw tremor and related TMD as manifestations of PD. When rigidity and jaw tremor are identified in TMD patients, a referral to the neurology department should be considered for further evaluation of PD.

All subgroups in both Study I and Study II did not show consistent results. This study initially started with a large set of population data. However, the number of subgroups was decreased due to the matching procedure based on the variable factors. The reduced number of subgroups may have led to the unreliable and insufficient results of subgroup analyses in both studies (Table 1 and Table 2).

This study has a few strengths. First, in this study, two different studies were performed to demonstrate the mutual association between TMD and PD. Second, this study consisted of a large patient cohort (*n* = 22,275 and 30,380 for Study I and II, respectively). Moreover, participants with TMD and PD were followed up for a maximum of 13 years. Third, the Korean National Health Insurance Service Health Screening Cohort dataset is a large, national dataset that is representative of the Korean population. The survey was exclusive to the Korean population. Fourth, both TMD and PD are associated with reduced quality of life, and a better understanding of these two conditions could potentially hold clinical significance for future treatment. Fifth, this study adjusted various influential factors to reduce surveillance bias [46]. Lastly, this study was performed by well-trained clinicians such as dentists and medical doctors and included general health examination and laboratory evaluations [47]. A previous study employed a questionnaire yielding subjective data that could result in recall bias [35.] These results indicate that medical doctors could prevent the aggravation of TMD in PD patients by examining abnormal jaw movement such as bruxism and discussing this condition with dentists. Likewise, dentists should be cautious of jaw tremor or rigidity as an initial sign of PD. If the symptoms are confirmed, then clinicians should refer the patients to the neurology department for further evaluation.

This study also has some limitations. First, the number of participants included in the subgroups was reduced through the matching procedure, rendering the subgroup analyses less optimal. Although this study started with 514,866 participants, there was a decrease in the number of participants found to have a diagnosis of both TMD and PD. In addition, this study conducted the subgroup analysis as the sensitivity analysis of the main result. In the subgroup analyses, some groups showed a statistically significant difference, while others did not. This study could not explain the difference between these groups using the current data. This might be due to differences in their behavior, such as diet pattern, exercise, and general health status. Second, this study attempted to adjust as many confounding factors as possible. However, it was impossible to adjust for all systemic factors that were not included in the large set of population data [48].

## 5. Conclusions

This study demonstrated that patients with TMD have a significantly higher risk of developing PD and that those with PD have a significantly higher risk of developing TMD. Clinicians should be cautious of jaw tremor or rigidity as an early sign of PD. If these symptoms are confirmed, then clinicians should refer the patients to the neurology department for further evaluation.

## Figures and Tables

**Table 1:** General characteristics of the participants.

Characteristics		Study I			Study II		
		TMD	Control I	*p*-Value	PD	Control II	*p*-Value
Age (*n*, %)				1.000			1.000
	40–44	130 (2.9)	520 (2.9)		8 (0.1)	32 (0.1)	
	45–49	437 (9.8)	1748 (9.8)		75 (1.2)	300 (1.2)	
	50–54	697 (15.7)	2788 (15.7)		234 (3.9)	936 (3.9)	
	55–59	718 (16.1)	2872 (16.1)		361 (5.9)	1444 (5.9)	
	60–64	634 (14.2)	2536 (14.2)		654 (10.8)	2616 (10.8)	
	65–69	699 (15.7)	2796 (15.7)		1014 (16.7)	4056 (16.7)	
	70–74	603 (13.5)	2412 (13.5)		1431 (23.6)	5724 (23.6)	
	75–79	376 (8.4)	1504 (8.4)		1396 (23.0)	5584 (23.0)	
	80–84	130 (2.9)	520 (2.9)		721 (11.9)	2884 (11.9)	
	85+	31 (0.7)	124 (0.7)		182 (3.0)	728 (3.0)	
Sex (*n*, %)				1.000			1.000
	Males	1903 (42.7)	7612 (42.7)		2858 (47.0)	11,432 (47.0)	
	Females	2552 (57.3)	10,208 (57.3)		3218 (53.0)	12,872 (53.0)	
Income (*n*, %)				1.000			1.000
	1 (lowest)	699 (15.7)	2796 (15.7)		1151 (18.9)	4604 (18.9)	
	2	572 (12.8)	2288 (12.8)		672 (11.1)	2688 (11.1)	
	3	717 (16.1)	2868 (16.1)		825 (13.6)	3300 (13.6)	
	4	929 (20.9)	3716 (20.9)		1148 (18.9)	4592 (18.9)	
	5 (highest)	1538 (34.5)	6152 (34.5)		2280 (37.5)	9120 (37.5)	
Region of residence (*n*, %)				1.000			1.000
	Urban	1822 (40.9)	7288 (40.9)		2252 (37.1)	9008 (37.1)	
	Rural	2633 (59.1)	10,532 (59.1)		3824 (62.9)	15,296 (62.9)	
Obesity ^†^ (*n*, %)				<0.001 *			0.651
	Underweight	130 (2.9)	469 (2.6)		254 (4.2)	922 (3.8)	
	Normal	1731 (38.9)	6248 (35.1)		2165 (35.6)	8771 (36.1)	
	Overweight	1280 (28.7)	4808 (27.0)		1581 (26.0)	6389 (26.3)	
	Obese I	1219 (27.4)	5710 (32.0)		1883 (31.0)	7452 (30.7)	
	Obese II	95 (2.1)	585 (3.3)		193 (3.2)	770 (3.2)	
Smoking status (*n*, %)				<0.001 *			<0.001 *
	Non-smoker	3416 (76.7)	13,410 (75.3)		4789 (78.8)	18,269 (75.2)	
	Past smoker	526 (11.8)	1900 (10.7)		670 (11.0)	2897 (11.9)	
	Current smoker	513 (11.5)	2510 (14.1)		617 (10.2)	3138 (12.9)	
Alcohol consumption (*n*, %)				0.605			<0.001 *
	<1 time a week	3180 (71.4)	12,650 (71.0)		4746 (78.1)	17,613 (72.5)	
	≥1 time a week	1275 (28.6)	5170 (29.0)		1330 (21.9)	6691 (27.5)	
Systolic blood pressure (*n*, %)							0.194
	<120 mmHg	1480 (33.2)	5296 (29.7)	<0.001 *	1384 (22.8)	5458 (22.5)	
	120–139 mmHg	2168 (48.7)	8566 (48.1)		2877 (47.4)	11,814 (48.6)	
	≥140 mmHg	807 (18.1)	3958 (22.2)		1815 (29.9)	7032 (28.9)	
Diastolic blood pressure (*n*, %)				<0.001 *			0.396
	<80 mmHg	2264 (50.8)	8377 (47.0)		2656 (43.7)	10,659 (43.9)	
	80–89 mmHg	1542 (34.6)	6331 (35.5)		2188 (36.0)	8897 (36.6)	
	≥90 mmHg	649 (14.6)	3112 (17.5)		1232 (20.3)	4748 (19.5)	
Fasting blood glucose (*n*, %)				<0.001 *			<0.001 *
	<100 mg/dL	2928 (65.7)	11,222 (63.0)		3292 (54.2)	14,289 (58.8)	
	100–125 mg/dL	1201 (27.0)	5025 (28.2)		1918 (31.6)	7338 (30.2)	
	≥126 mg/dL	326 (7.3)	1573 (8.8)		866 (14.3)	2677 (11.0)	
Total cholesterol (*n*, %)				0.006 *			0.003 *
	<200 mg/dL	2387 (53.6)	9317 (52.3)		3414 (56.2)	13,189 (54.3)	
	200–239 mg/dL	1503 (33.7)	5912 (33.2)		1805 (29.7)	7774 (32.0)	
	≥240 mg/dL	565 (12.7)	2591 (14.5)		857 (14.1)	3341 (13.8)	
CCI score (*n*, %)				0.003 *			<0.001 *
	0	2988 (67.1)	12,106 (67.9)		2110 (34.7)	13,659 (56.2)	
	1	684 (15.4)	2599 (14.6)		1377 (22.7)	4383 (18.0)	
	2	410 (9.2)	1400 (7.9)		989 (16.3)	2652 (10.9)	
	3	169 (3.8)	766 (4.3)		652 (10.7)	1555 (6.4)	
	≥4	204 (4.6)	949 (5.3)		948 (15.6)	2055 (8.5)	
PD		47 (1.1)	132 (0.7)	0.036 *	6076 (100.0)	0 (0.0)	<0.001 *
TMD		4445 (100.0)	0 (0.0)	<0.001 *	53 (0.9)	145 (0.6)	0.017 *

Abbreviations: TMD, temporomandibular joint disorder; PD, Parkinson’s disease; CCI, Charlson comorbidity index. * Chi-square test was analyzed, significance at *p* < 0.05. ^†^ Obesity (BMI, body mass index, kg/m^2^) was categorized as <18.5 (underweight), ≥18.5 to <23 (normal), ≥23 to <25 (overweight), ≥25 to <30 (obese I), and ≥30 (obese II).

**Table 2:** Odd ratios for TMD in PD and non-PD groups in Study I.

Characteristics	Odd Ratios (95% Confidence Interval) for TMD			
	Crude ^†^	*p*-Value	Adjusted ^†,‡^	*p*-Value
Total participants (*n* = 22,275)				
PD	1.43 (1.02–2.01)	0.036 *	1.43 (1.02–2.00)	0.040 *
Non-PD	1.00		1.00	
Age < 60 years old, males (*n* = 4275)				
PD	6.00 (1.00–35.91)	0.050 *	5.68 (0.94–34.37)	0.059
Non-PD	1.00		1.00	
Age < 60 years old, females (*n* = 5635)				
PD	2.67 (0.45–16.06)	0.282	3.03 (0.50–18.40)	0.228
Non-PD	1.00		1.00	
Age ≥ 60 years old, males (*n* = 5240)				
PD	1.17 (0.64–2.14)	0.609	1.15 (0.63–2.10)	0.658
Non-PD	1.00		1.00	
Age ≥ 60 years old, females (*n* = 7125)				
PD	1.43 (0.92–2.21)	0.109	1.40 (0.90–2.17)	0.138
Non-PD	1.00		1.00	
High income (*n* = 9940)				
PD	1.85 (1.11–3.08)	0.018 *	1.80 (1.08–3.00)	0.025 *
Non-PD	1.00		1.00	
Low income (*n* = 12,335)				
PD	1.20 (0.76–1.88)	0.440	1.20 (0.76–1.89)	0.429
Non-PD	1.00		1.00	
Urban living (*n* = 9110)				
PD	1.58 (0.91–2.73)	0.105	1.56 (0.90–2.72)	0.114
Non-PD	1.00		1.00	
Rural living (*n* = 13,165)				
PD	1.36 (0.89–2.08)	0.160	1.35 (0.88–2.07)	0.167
Non-PD	1.00		1.00	

Abbreviation: TMD, temporomandibular joint disorder; PD, Parkinson’s disease; CCI, Charlson comorbidity index. * Conditional logistic regression model, Significance at *p* < 0.05. ^†^ Models stratified by age, sex, income, and region of residence. ^‡^ Models adjusted for obesity, smoking, alcohol consumption, systolic blood pressure, diastolic blood pressure, fasting blood glucose, total cholesterol, and CCI scores.

**Table 3:** Odd ratios for PD in TMD and non-TMD groups in Study II.

Characteristics	Odd Ratios (95% Confidence Interval) for PD			
	Crude ^†^	*p*-Value	Adjusted ^†,‡^	*p*-Value
Total participants (*n* = 30,380)				
TMD	1.47 (1.07–2.01)	0.018 *	1.56 (1.13–2.15)	0.007 *
Non-TMD	1.00		1.00	
Age < 70 years old, males (*n* = 5770)				
TMD	1.60 (0.50–5.12)	0.426	2.16 (0.67–7.00)	0.198
Non-TMD	1.00		1.00	
Age < 70 years old, females (*n* = 5960)				
TMD	1.34 (0.63–2.85)	0.454	1.33 (0.61–2.86)	0.472
Non-TMD	1.00		1.00	
Age ≥ 70 years old, males (*n* = 8520)				
TMD	1.37 (0.76–2.46)	0.298	1.40 (0.77–2.54)	0.266
Non-TMD	1.00		1.00	
Age ≥ 70 years old, females (*n* = 10,130)				
TMD	1.57 (0.99-2.50)	0.057	1.68 (1.04–2.70)	0.033 *
Non-TMD	1.00		1.00	
High income (*n* = 13,240)				
TMD	1.59 (0.98–2.59)	0.060	1.66 (1.01–2.73)	0.046 *
Non-TMD	1.00		1.00	
Low income (*n* = 17,140)				
TMD	1.38 (0.91–2.10)	0.127	1.49 (0.98–2.28)	0.062
Non-TMD	1.00		1.00	
Urban living (*n* = 11,260)				
TMD	1.31 (0.74–2.31)	0.352	1.40 (0.78–2.48)	0.257
Non-TMD	1.00		1.00	
Rural living (*n* = 19,120)				
TMD	1.55 (1.06–2.27)	0.025 *	1.65 (1.12–2.43)	0.011 *
Non-TMD	1.00		1.00	

Abbreviation: TMD, Temporomandibular joint disorder; PD, Parkinson’s disease; CCI, Charlson comorbidity index. * Conditional logistic regression model, significance at *p* < 0.05. ^†^ Models stratified by age, sex, income, and region of residence. ^‡^ Models adjusted for obesity, smoking, alcohol consumption, systolic blood pressure, diastolic blood pressure, fasting blood glucose, total cholesterol, and CCI scores.

## Data Availability

Data was obtained from Korean National Health Insurance Service and are available with the permission of Korean National Health Insurance Service.

## Supplementary Materials

The following are available online at https://www.mdpi.com/article/10.3390/brainsci11060747/s1, Table S1: Subgroup analyses of odd ratios for TMD in PD and non-PD in Study I; Table S2: Subgroup analyses of odd ratios for PD in TMD and non-TMD groups in Study II.
