# Supplementary material for: Association between Temporomandibular Joint Disorder and Parkinson’s Disease
Source: Brain Sci. 2021 Jun 4;11(6):747. doi: 10.3390/brainsci11060747 (PMC8229075; doi:10.3390/brainsci11060747)
Supplement: Supplementary file 1 [file brainsci-11-00747-s001.zip › brainsci-1241773-supplementary.pdf]

Table S1 Subgroup analyses of odd ratios for TMD in PD and non-PD in study I

Characteristics	Odd ratios (95% confidence interval) for TMD			
	Crude	P-value	Adjusted†	P-value
BMI < 23 (n = 8,578)				
PD	1.36 (0.82-2.25)	0.235	1.40 (0.84-2.32)	0.196
Non-PD	1.00		1.00	
BMI ≥ 23 (n = 13,697)				
PD	1.47 (0.94-2.30)	0.092	1.51 (0.96-2.37)	0.072
Non-PD	1.00		1.00	
Nonsmoker (n = 16,826)				
PD	1.50 (1.05-2.14)	0.024*	1.53 (1.07-2.19)	0.019*
Non-PD	1.00		1.00	
Past smoker and current smoker (n = 5,449)				
PD	0.89 (0.30-2.63)	0.838	0.92 (0.31-2.72)	0.875
Non-PD	1.00		1.00	
Alcohol consumption < 1 time a week (n = 15,830)				
PD	1.39 (0.96-2.02)	0.081	1.42 (0.97-2.06)	0.068
Non-PD	1.00		1.00	
Alcohol consumption ≥ 1 time a week (n = 6,445)				
PD	1.59 (0.74-3.45)	0.238	1.59 (0.73-3.46)	0.244
Non-PD	1.00		1.00	
Systolic blood pressure < 140 mmHg and diastolic blood pressure < 90 mmHg (n = 16,594)				
PD	1.49 (0.99-2.24)	0.059	1.49 (0.98-2.24)	0.060
Non-PD	1.00		1.00	
Systolic blood pressure ≥ 140 mmHg or diastolic blood pressure ≥ 90 mmHg (n = 5,681)				
PD	1.43 (0.80-2.55)	0.232	1.45 (0.81-2.61)	0.213
Non-PD	1.00		1.00	
Fasting blood glucose < 100 mg/dL (n = 14,150)				

PD	1.18 (0.74-1.88)	0.496	1.18 (0.74-1.90)	0.489
Non-PD	1.00		1.00	
Fasting blood glucose ≥ 100 mg/dL (n = 8,125)				
PD	1.83 (1.13-2.96)	0.013*	1.85 (1.14-3.00)	0.013*
Non-PD	1.00		1.00	
Total cholesterol < 200 mg/dL (n = 11,704)				
PD	1.38 (0.88-2.16)	0.164	1.38 (0.88-2.17)	0.165
Non-PD	1.00		1.00	
Total cholesterol ≥ 200 mg/dL (n = 10,571)				
PD	1.50 (0.91-2.47)	0.116	1.57 (0.95-2.61)	0.080
Non-PD	1.00		1.00	

Abbreviation: CCI, Charlson Comorbidity Index;

* Logistic regression model, Significance at $P < 0.05$

† Models adjusted for obesity, smoking, alcohol consumption, systolic blood pressure, diastolic blood pressure, fasting blood glucose, total cholesterol and CCI scores.

Table S2 Subgroup analyses of odd ratios for PD in TMD and non-TMD groups in study II

Characteristics	Odd ratios (95% confidence interval) for PD			
	Crude	P-value	Adjusted†	P-value
BMI < 23 (n = 12,112)				
TMD	1.98 (1.28-3.08)	0.002*	2.04 (1.31-3.19)	0.002*
Non-TMD	1.00		1.00	
BMI ≥ 23 (n = 18,268)				
TMD	1.10 (0.69-1.74)	0.702	1.12 (0.70-1.79)	0.628
Non-TMD	1.00		1.00	
Nonsmoker (n = 23,058)				
TMD	1.54 (1.08-2.20)	0.017*	1.56 (1.09-2.24)	0.015*
Non-TMD	1.00		1.00	
Past smoker and current smoker (n = 7,322)				
TMD	1.24 (0.61-2.49)	0.553	1.26 (0.62-2.56)	0.518
Non-TMD	1.00		1.00	
Alcohol consumption < 1 time a week (n = 22,359)				
TMD	1.34 (0.91-1.96)	0.135	1.34 (0.91-1.98)	0.138
Non-TMD	1.00		1.00	
Alcohol consumption ≥ 1 time a week (n = 8,021)				
TMD	1.91 (1.09-3.35)	0.024*	1.92 (1.09-3.38)	0.024*
Non-TMD	1.00		1.00	
Systolic blood pressure < 140 mmHg and diastolic blood pressure < 90 mmHg (n = 20,446)				
TMD	1.43 (1.00-2.05)	0.053	1.45 (1.00-2.09)	0.048*
Non-TMD	1.00		1.00	
Systolic blood pressure ≥ 140 mmHg or diastolic blood pressure ≥ 90 mmHg (n = 9,934)				
TMD	1.63 (0.85-3.13)	0.138	1.73 (0.90-3.33)	0.103

Non-TMD	1.00		1.00	
Fasting blood glucose < 100 mg/dL (n = 17,581)				
TMD	1.59 (1.06-2.38)	0.026*	1.62 (1.07-2.44)	0.022*
Non-TMD	1.00		1.00	
Fasting blood glucose ≥ 100 mg/dL (n = 12,799)				
TMD	1.33 (0.80-2.19)	0.268	1.36 (0.82-2.27)	0.239
Non-TMD	1.00		1.00	
Total cholesterol < 200 mg/dL (n = 16,603)				
TMD	1.18 (0.76-1.84)	0.455	1.22 (0.78-1.90)	0.383
Non-TMD	1.00		1.00	
Total cholesterol ≥ 200 mg/dL (n = 13,777)				
TMD	1.89 (1.20-2.98)	0.006*	1.93 (1.21-3.06)	0.006*
Non-TMD	1.00		1.00	

Abbreviation: CCI, Charlson Comorbidity Index;

* Logistic regression model, Significance at $P < 0.05$

† Models adjusted for obesity, smoking, alcohol consumption, systolic blood pressure, diastolic blood pressure, fasting blood glucose, total cholesterol, and CCI scores.
